# Embryogenic potential and expression of embryogenesis-related genes in conifers are affected by treatment with a histone deacetylase inhibitor

**DOI:** 10.1186/1753-6561-5-S7-P151

**Published:** 2011-09-13

**Authors:** Daniel Uddenberg, Silvia Valladares, Malin Abrahamsson, Jens F Sundström, Annika Sundås-Larsson, Sara von Arnold

**Affiliations:** 1 Uppsala Biocenter, Department of Plant Biology and Forest Genetics, Swedish University of Agricultural Sciences (SLU), Uppsala, Box 7080, SE-750 07, Sweden; 2 Evolutionary Biology Center, Physiological Botany, Uppsala University, Uppsala, SE-752 36, Sweden; 3 Department of Plant Physiology, Instituto de Investigaciones Agrobiologicas de Galicia (CSIC), Santiago de Compostela, Apartado 122, 15780, Spain

## Background

Somatic embryogenesis is a useful method to propagate conifers vegetatively. In many coniferous species embryogenic cultures can be established from zygotic embryos, while the embryogenic potential decreases during germination. Embryo formation is thought to require a signal that induces a somatic cell to dedifferentiate and gain embryogenic potential as well as the expression of an appropriate cellular environment for the response of the inductive signal. In *Arabidopsis thaliana* (*Arabidopsis*) *LEAFY COTYLEDON* (*LEC*) genes are expressed during the embryonic stage but must be repressed to allow germination [1]. Treatment with the histone deacetylase inhibitor trichostatin A (TSA) causes de-repression of *LEC* genes. In addition, ectopic post-embryonic expression of *LEC1* is sufficient to induce differentiation of embryo-like structures from vegetative cells [2]. *ABSCISIC ACID3* (*ABI3*) and its maize (*Zea mays*) orthologue *VIVIPAROUS1* (*VP1*) act together with the *LEC* genes to promote embryo maturation [3]. Knowledge about the molecular mechanisms underlying embryogenic competence in conifers is largely uncharacterized, although this is the foundation for propagation of conifers through somatic embryos. However, we have recently shown that TSA-treatment affects both the embryogenic potential and the expression of embryogenesis-related genes in Norway spruce [4].

## Materials and methods

Conifer sequences of LEC1-type *HAP3* and *ABI3/VP1* homologues were retrieved from public databases and isolated in Norway spruce (*Picea abies*) and Scots Pine (*Pinus sylvestris*). Phylogenetic analyses were done on nucleotide alignments of conserved domains made using Baysian inference (MrBayes) and maximum parsimony (PAUP*). Embryogenic cell lines 06.28.05 of Norway spruce and 12:12 of Scots pine were used in this study. To analyze the effect of TSA (Sigma-Aldrich) during maturation and germination of somatic embryos of Norway spruce, the growth media were supplemented with 10μM TSA. Expression levels of the conifer *HAP3a* and *VP1* genes were assessed using quantitative real-time PCR.

## Results and discussion

We isolated two conifer LEC1-type HAP3 genes, *HAP3A* and *HAP3B*, from Norway spruce and Scots pine*.* A comparative phylogenetic analysis of plant HAP3 genes suggests that *HAP3A* and *HAP3B* are paralogous genes originating from a duplication event in the conifer lineage. The angiosperm ABI3/*VP1* genes belong to the plant specific B3 gene family and phylogenetic relationship position the conifer homologs closest to ABI3 and VP1. The expression of *HAP3A* is high during early embryo development but decreases during late embryogeny, while the expression of *VP1* is initially low and increases during late embryogeny, in both Norway spruce and Scots pine. The expression levels for both genes are similar during somatic and zygotic embryogenesis. When embryogenic cultures of Norway spruce were exposed to TSA during embryo maturation, maturation was arrested and the expression levels of *PaHAP3A* and *PaVP1* were maintained. Furthermore, when germinating somatic embryos of Norway spruce were treated with TSA, the germination progression was partially inhibited and the embryogenic potential was maintained at a similar level as embryos before germination.

## Conclusions

Taken together, our results suggest that important regulators of embryogenesis are conserved between angiosperms and gymnosperms, and, assuming that TSA affects histone acetylation in conifers, our results indicate a connection between between chromatin structure and expression of embryogenesis-related genes in conifers.
